# The Identification of Biochanin A as a Potent and Selective β-Site App-Cleaving Enzyme 1 (Bace1) Inhibitor

**DOI:** 10.3390/nu8100637

**Published:** 2016-10-14

**Authors:** Kumju Youn, Ji-Hyun Park, Jinhyuk Lee, Woo-Sik Jeong, Chi-Tang Ho, Mira Jun

**Affiliations:** 1 Department of Food Science and Nutrition, Dong-A University, Busan 604-714, Korea; kjyoun@hanmail.net (K.Y.); jhpark9043@gmail.com (J.-H.P.); 2 Korean Bioinformation Center, Korea Research Institute of Bioscience and Biotechnology, Daejeon 305-806, Korea; mack97hyuk@gmail.com; 3 Department of Bioinformatics, University of Sciences and Technology, Daejeon 305-350, Korea; 4 Department of Food & Life Science, College of Biomedical Science & Engineering, Inje University, Gimhae 621-749, Korea; 5 Department of Food Science, Rutgers University, New Brunswick, NJ 08901, USA; ho@aesop.rutgers.edu; 6 Institute of Convergence Bio-Health (ICBH), Dong-A University, 32, Daeshingongwon-Ro, Seo-Gu, Busan 602-715, Korea

**Keywords:** Alzheimer’s disease, β-secretase (BACE1), β-amyloid peptide (Aβ), biochanin A

## Abstract

Beta-site amyloid precursor protein cleaving enzyme 1 (BACE1) is the enzyme involved in the abnormal production of the amyloidogenic peptide Aβ, one of the major causes of histological hallmarks of Alzheimer’s disease (AD). Thus, BACE1 represents a key target protein in the development of new potential target for the prevention and treatment of AD. In this study, in vitro anti-AD activity of biochanin A, a dietary isoflavone found in legumes and most notably red clover, were evaluated via human recombinant BACE1 inhibition assay, as well as enzyme kinetic and molecular docking predictions. Enzyme-based assays revealed that biochanin A exhibited a non-competitive inhibitory effect on BACE1 with an IC_50_ value of 28 μM and a *Ki* of 43 μM. In addition, docking simulation results demonstrated that ASN37, SER35, SER36, TRP76, and ARG128 residues of BACE1 interacted with biochanin A. Moreover, the binding energy of biochanin A was negative (−8.4 kcal/mol), indicating that it might potentiate a strong binding between the compound and the allosteric site of BACE1, resulting in further effective BACE1 inhibition. The present novel findings raise the possibility that biochanin A may be used as a preventative, developed into a therapeutic agent for AD, or both.

## 1. Introduction

Alzheimer’s disease (AD) is a neurodegenerative disorder presenting with symptoms such as memory loss and disruption in judgment, reasoning, and emotional stability. The predominant pathological hallmarks of the disease are amyloid plaques, i.e., extracellular deposits of polymerized amyloid-β peptides (Aβ), and intracellular aggregates of misfolded tau protein [1,2]. Much of AD research have been focused on the amyloid cascade hypothesis, which states that Aβ, a proteolytic derivative of the large transmembrane protein amyloid precursor protein (APP), plays an early role in the pathogenesis of AD [3]. The produced Aβ is normally secreted, but also can accumulate and form insoluble aggregates [4,5].

β-Secretase and γ-secretase have been identified as the two key proteases responsible for the processing of the membrane-bound APP to the 40/42 residue Aβ [6]. β-APP cleaving enzyme 1 (BACE1) is the main form of β-secretase that cleaves APP to generate Aβ [7]. The unique and essential role of BACE1 for the generation of Aβ has been convincingly shown in mice where both alleles have been ablated by genetic means. These mice neither formed Aβ, nor cleaved APP at the known BACE1 positions [8]. It has moreover been demonstrated that crossing BACE1 knockout mice with APP-transgenic mice leads to inhibition of brain amyloid formation [9,10]. Knockout of BACE1 gene (not BACE2) was found to lead to hypomyelination through interference with the processing of neuregulin-1; however, a recent report demonstrated that BACE1 inhibitors did not affect the neuregulin-1 processing significantly, ensuring the safety of BACE1 inhibition in AD therapy, to some extent [11]. Taken together, all these findings make BACE1 a prime target for anti-amyloid therapy.

Plant isoflavone biochanin A can be found in legumes, abundantly in red clover, and in commercially available extracts [12]. In contrast to its unmethylated analog genistein, biochanin A is not present in soy in significant quantity, but it can be found in many other legumes and peanuts. Its chemical structure resembles that of estrogen; hence, it is able to show agonistic and antagonistic interactions with the estrogen receptor [13]. Over the past few years, the research on the isoflavones has mostly been focused on genistein, and the limited number of studies have been performed on the role of biochanin A. Interestingly, in our ongoing research of exploring natural BACE1 inhibitor, biochanin A exhibited significant BACE1 suppressing property compared with the other isoflavones including genistein. Furthermore, previous studies regarding on biochanin A displayed marked neuroprotective properties [14,15,16,17]. Biochanin A attenuated Aβ_25–35_-induced PC12 cell injury and apoptosis via preventing mitochondrial dysfunction [14]. The compound protected dopaminergic neurons against LPS-induced damage through inhibition of microglia activation and production of proinflammatory factors [15]. Red clover extract had neuroprotective efficacy in H_2_O_2_-induced human cortical cells [16]. In addition, the administration of biochanin A reduced the increased level of adrenalin and dopamine, a sign of dementia in scopolamine-treated mice [16]. Despite these neuroprotective findings, there is no detailed study on the inhibition and molecular interaction between biochanin A and BACE1, a vital target for AD prevention.

In the present study, an approach to develop biochanin A as a potent anti-AD drug candidates was proposed through the biological evaluation of BACE 1 activity and in silico molecular docking study of human BACE1.

## 2. Materials and Methods

### 2.1. General

Biochanin A (5,7-dihydroxy-3-(4-methoxyphenyl)chromen-4-one) (≥97% purity) and resveratrol (used as a positive control) was purchased from Sigma-Aldrich (St. Louis, MO, USA). Ethanol (>99.8%) was also purchased from Sigma-Aldrich. A BACE1 assay kit was purchased from Pan Vera (Madison, WI, USA). α-Secretase (TACE) and substrates were obtained from R & D systems (Minneapolis, MN, USA). Trypsin, chymotrypsin, elastase, and their substrates were obtained from Sigma-Aldrich (St. Louis, MO, USA). Fluorescence and optical density were measured with a Bio-TEK ELISA microplate fluorescence reader FLx 800 and Bio-TEK ELx 808 (Bio-Tek, Winooski, VT, USA), respectively.

### 2.2. Enzymatic Assay of BACE1, TACE and Other Serine Proteases

The inhibitory property of biochanin A on BACE1 was evaluated by a fluorescence resonance energy transfer (FRET) assay (Pan Vera) with a recombinant baculovirus-expressed BACE1 and a specific substrate (Rh-EVNLDAEFK-Quencher) according to manufacturer instructions. Briefly, a mixture of human recombinant BACE1 (1.0 U/mL), the substrate (75 μM in 50 mM ammonium bicarbonate), and biochanin A dissolved in an assay buffer (50 mM sodium acetate, pH 4.5), was incubated for 60 min at 25 °C in well plates. The increase in fluorescence intensity produced by substrate hydrolysis was observed on a fluorescence microplate reader with excitation and emission wavelengths of 545 and 590 nm, respectively. The inhibition ratio was obtained using the following equation:

Inhibition (%) = [1 − (*S* − *S*_0_)/(*C* − *C*_0_)] × 100,

where *C* was the fluorescence of control (enzyme, assay buffer, and substrate) after 60 min of incubation, *C*_0_ was the fluorescence of control at time 0, *S* was the fluorescence of tested samples (enzyme, sample solution, and substrate) after 60 min of incubation, and *S*_0_ was the fluorescence of the tested samples at time 0.

TACE inhibitory activity was measured with a manual from the R & D system. A human recombinant TACE (0.1 ppm in 25 mM Tris buffer), the substrate (APP peptide YEVHHQKLV using EDANS/DABCYL), and biochanin A dissolved in an assay buffer, which were then combined and incubated for 60 min in the dark at 25 °C. The increase in fluorescence intensity produced by substrate hydrolysis was observed on a fluorescence microplate reader with excitation and emission wavelengths of 320 and 405 nm, respectively. The fluorescence intensity was measured in a microplate reader at excitation and emission wavelengths of 340 and 535 nm, respectively. 

Trypsin, chymotrypsin, and elastase were assayed according to the protocol described in [18]. The inhibitory activities of biochanin A towards trypsin, chymotrypsin, and elastase were measured using the spectrophotometric method. *N*-benzoyl-l-Arg-*p*NA, *N*-benzoyl-l-Tyr-*p*NA, and *N*-succinyl-Ala-Ala-Ala-*p*NA were used as substrates to assay the inhibition of trypsin, chymotrypsin, and elastase, respectively. Enzyme, Tris-HCl buffer (0.05 M, in 0.02 M CaCl_2_, pH 8.2), and biochanin A were incubated for 10 min at 25 °C then added substrate for 30 min at 37 °C. The absorbance was recorded at 410 nm. The inhibition ratio was obtained using the following equation:

Inhibition (%) = {[1 − (A − B)]/control} × 100,

where A was the absorbance of control (enzyme, assay buffer, and substrate) after 60 min of incubation, and A was the absorbance of tested samples (assay buffer and sample solution) after 60 min of incubation.

### 2.3. Kinetic Studies of Biochanin A against BACE1

To determine the kinetics of inhibition of BACE1 by biochanin A, we employed two complementary kinetic methods, a dixon plot [plot of 1/enzyme velocity (1/V) against inhibitor concentration (I)] and a Linwever-Burk plot [a plot of 1/enzyme velocity (1/V) versus 1/substrate concentration (1/[S])]. The modes of inhibition were determined by both methods, whereas the inhibitory constant (*Ki*) value was calculated from the Dixon plot. Michaelis constant (*K*_m_) and the maximum velocity (*V*_max_) values were determined by means of Lineweaver-Burk plots, using initial velocities obtained over a substrate concentration ranging from 250, 500, and 750 nM. Graphs were plotted by using Sigma plot software (Version 12.0, Systat software Inc., San Jose, CA, USA).

### 2.4. Molecular Docking Studies of Biochanin A

The 3D crystal structure of the BACE1 was obtained from the Protein Data Bank (PDB ID: 2WJO). Marvin was used for drawing, displaying, and characterizing the chemical structures (Marvin 5.11.4, 2012, ChemAxon [19]). Potential binding pocket residue in human BACE1 was identified using the Pck software (The Scripps Research Institute, La Jolla, CA, USA). Molecular docking simulation was performed using Autodock Vina (The Scripps Research Institute, La Jolla, CA, USA), which automatically calculates the grid maps [20]. Specifically, Autodock Vina has improved speed and accuracy of docking due to threading capabilities [21]. The used parameters were as follows: xyz center coordinates of the binding pocket residues; search space (15 Å) in each dimension; and generation number (10) of each binding pocket residue. All docking structures were ranked by the lowest energy.

### 2.5. Statistical Analysis

All experiments were performed in triplicate. Data of each experiments were expressed as mean ± SE. Significant differences were analyzed by Student’s *t*-test and Duncan’s multiple range tests using Statistical Analysis System (SAS) version 9.3.8 (SAS Inc., Cary, NC, USA).

## 3. Results

### 3.1. In Vitro BACE1 Inhibitory Activity of Biochanin A

**Figure 1:**
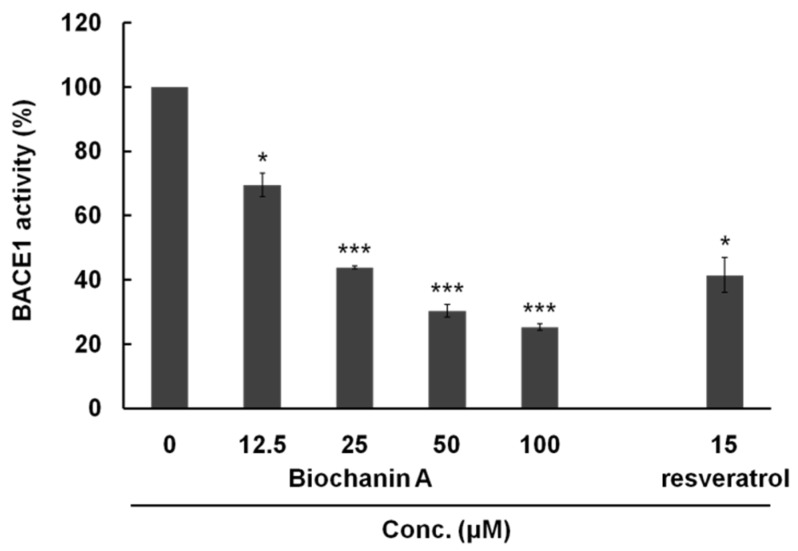
Concentration-dependent BACE1 inhibitory activity of biochanin A. Resveratrol was used as a positive control. Comparison of untreated and treated biochanin A is significantly different at * *p* < 0.05 and *** *p* < 0.001.

We investigated whether biochanin A has BACE1 inhibitory activity by an in vitro BACE1 activity assay. BACE1 activity was found to decrease in a dose-dependent manner to 69.54% ± 3.62%, 43.89% ± 0.52%, 30.31% ± 1.97%, and 25.31% ± 1.02% by biochanin A at 12.5, 25, 50, and 100 μM, respectively (Figure 1). Statistically, compared with the biochanin A untreated group, biochanin A exhibited significant differences at all the tested concentrations (*p* < 0.05 and *p* < 0.001), with an IC_50_ of 28 μM.

### 3.2. Kinetic Parameters of Biochanin A

**Figure 2:**
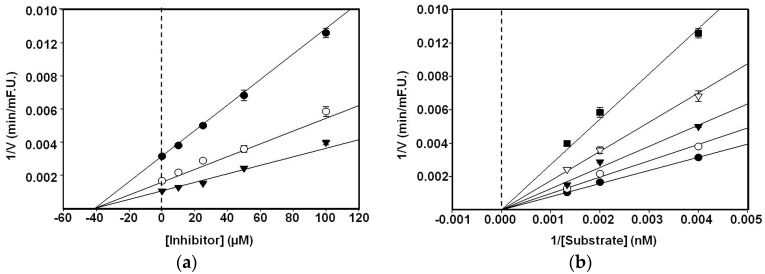
Dixon plot for BACE1 inhibition by biochanin A (**a**). Biochanin A was in the presence of different concentration of substrate: 250 nM (●); 500 nM (○); 750 nM (▼); Lineweaver–Burk plots for BACE1 inhibition of biochanin A (**b**). BACE1 inhibition was analyzed in the presence of different concentrations of biochanin A as follows: 0 μM (●); 12.5 μM (○); 25 μM (▼); 50 μM (▽); 100 μM (■).

In an attempt to explain the mode of enzymatic inhibition of biochanin A, kinetic analyses were performed using different concentrations of the substrate (250, 500, and 750 nM) and an inhibitor. Concentrations of biochanin A (0, 12.5, 25, 50, and 100 μM) were also selected for this study. The reaction mixture consisted of the same, aforementioned BACE1 assay method. In non-competitive inhibition, the *x*-axis implies –*Ki* when 1/V = 0. As shown in Figure 2, the line for various concentrations of the substrates in the Dixon plots and those for biochanin A in the Lineweaver–Burk plot showed the same *x*-intercept, indicating non-competitive inhibition, with *Ki* values of 43 μM, suggesting that they might bind to either a β-secretase sub-site or to another regulatory site. It implies that biochanin A reduces the activity of the BACE1 and binds equally well to the enzyme whether or not it has already bound the substrate.

### 3.3. Biochanin A Interacts with BACE1 in Docking Analysis

**Figure 3:**
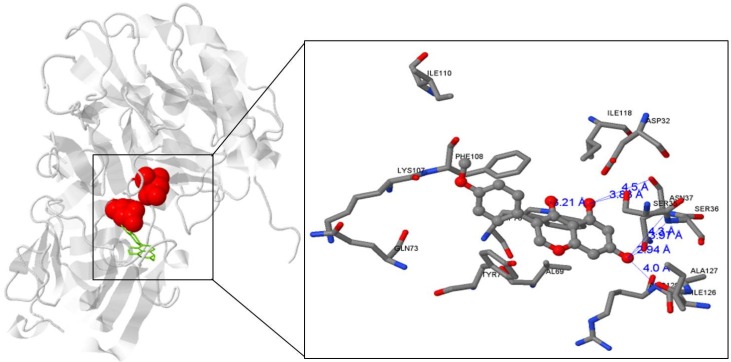
In silico docking poses for biochanin A. The complete view of the docking poses and the close up figure of biochanin A docking modes (**left**). Human BACE1 is expressed as a gray solid ribbon diagram and biochanin A as a green representation. Asp32 and Asp228 are the active catalytic center residues marked in red. Hydrogen bond interactions between biochanin A and human BACE1 residues are displayed as a blue line (**right**). The structural ligands were performed by AutoDock Vina.

The molecular docking models of biochanin A (green color) is illustrated in Figure 3. The corresponding ligand interactions of biochanin A in the BACE1 excluding active catalytic centers of BACE1 (Asp32 and Asp228) consisted of five hydrogen bonding interactions between the ASN37, SER35, SER36, TRP76, and ARG128 residues of the enzyme. The hydroxyl moiety of biochanin A acted simultaneously as a hydrogen donor and acceptor. The nitrogen atoms of SER 36, ASN37 36, TRP 76, and ARG 128 in BACE1 are hydrogen bonded to the oxygen atoms of biochanin A (distance: 3.97, 4.30, 3.21, and 4.00 Å, respectively). In contrast, the two oxygen atoms of biochanin A in C5 and C7 of A ring donated hydrogen to the oxygen atoms of ASN37 (distance: 4.50 Å and 2.94 Å). In addition, the lowest energy conformations of the most proposed complexes of biochanin A with BACE1 was −8.4 kcal/mol. Our analysis showed that biochanin A might bind to BACE1 allosteric sites of BACE1 through hydrogen bond interactions and thus has the potential to act as a non-competitive inhibitor of the enzyme.

### 3.4. Inhibition of TACE and Other Proteases Structurally Related to BACE1

The molecular culprit in AD is the Aβ, which is derived through sequential cleavage by the two proteases β- and γ-secretase. In contrast, TACE cleaves APP within the Aβ domain, thus prevents Aβ generation. In addition, the closest structural relatives to BACE 1 are the aspartyl proteases trypsin, chymotrypsin, and elastase. To determine the enzyme specificity and selectivity, the inhibitory property of the biochanin A against TACE and other serine proteases such as chymotrypsin, trypsin, and elastase was investigated (Table 1). Biochanin A did not display any statistical significance of inhibition against TACE and other serine proteases when compared with that of BACE 1, which suggests that biochanin A is a selective and specific inhibitor of BACE1.

## 4. Discussion

In this study, the impact of biochanin A on BACE1 inhibition was investigated. The current failure rate of finding BACE1 inhibitors has driven research interests towards the search for alternative small molecules with therapeutic potential for reducing risk or slowing progression of dementia associated with AD. Given the brain localization of the target, inhibitors are required to be of low molecular weight and should have reduced susceptibility to P-glycoprotein (Pgp)-mediated efflux transport at the blood–brain barrier (BBB) [22]. To effectively fulfill this aim, in recent years, there has been an obvious shift towards the exploration of non-peptidic inhibitors from natural sources [23]. Hopeful finding is that phytoestrogen biochanin A with 284.26 molar mass is demonstrated to be an inhibitor of Pgp, a major efflux transporter protein in human intestinal Caco-2 cells or MCF-7, which makes its way one step forward to AD prevention [24,25].

Biochanin A was proved to possess anti-angiogenic and neuroprotective properties. Administration of 50 mg/kg biochanin A significantly reduced tumor size and edema in the brains of male Fisher rats [26]. In addition, the administration of biochanin A for 14 successive days attenuated scopolamine-induced rise in oxidative damage as indicated by the reduced TBARS and increased GSH levels [17]. Furthermore, biochanin A was identified to be highly effective on human and rodent glioma cells while exhibiting a low-toxic profile on healthy brain tissue, indicating that biochanin A can be regarded as safe with respect to adverse reaction as it does not induce cell death in healthy brain tissue [26].

In vitro and in vivo studies have shown that biochanin A is metabolized to genistein by a demethylation pathway [27]. After demethylation, biochanin A and its metabolite genistein undergo glucuronidation and sulfation. The oxidative metabolism of biochanin A and genistein by cytochrome P450 enzymes has been observed when biochanin A and genistein are incubated with human or rat liver microsomes. The metabolites are mainly hydroxylated products such as 3′-, 6-, or 8-hydroxy biochanin A or genistein. 

Bioavailability of biochanin A is still controversial. Moon et al. (2006) suggested that the bioavailability of biochanin A was only 2.6% after a 5 mg/kg dose and 1.2% after a 50 mg/kg dose of biochanin A, which may be due to extensive first-pass metabolism by phase I oxidative metabolism and phase II glucuronidation and/or sulfation in the intestine as well as in the liver [28]. However, bioavailability may be improved by a three-fold increase by the combinations of flavonoids such as quercetin and EGCG, which speculated that both the inhibition of phase 2 metabolism (conjugation by UGT and SULT) and ABC efflux transporters might have contributed to this effect [29]. Although biochanin A can be metabolized to genistein conjugates, its biological effects observed in vivo are not exactly identical to those of genistein. Biochanin A, in contrast to genistein, can significantly suppress the tumor growth of human gastrointestinal cancer cells HCS-45M2 and HSC-41E6 transplanted in athymic nude mice [30]. Hence, it can be suggested that biochanin A and its metabolites exert significant and unique in vivo effects, other than those derived from genistein. Therefore, there are no adequate data to affirm or negate that biochanin A might be directly absorbed and metabolized to genistein conjugates and that these compounds might act as anti-dementia agents in the brain. We will further investigate the possibility of their bioavailability and permeability through the BBB in the near future. Although further and detailed explanation has yet to be given, it is still meaningful that biochanin A exerted significant and specific inhibitory properties against in vitro BACE1. 

## 5. Conclusions

Our findings indicated that biochanin A effectively inhibited the activity of BACE1 in a dose-dependent and non-competitive manner. Furthermore, our results provided important mechanistic insights into the binding mechanism of this compound to BACE1. Taken together, this study reveals that the biochanin A contained herein would clearly play a beneficial role in the development of therapeutic and preventive agents of AD and suggest potential guidelines for designing BACE1 selective inhibitors.

## Figures and Tables

**Table 1:** α-Secretase (TACE), trypsin, chymotrypsin, and elastase inhibition (%) ^1,2^ of biochanin A.

Biochanin A (μM)	TACE	Trypsin	Chymotrypsin	Elastase
50	18.33 ± 2.14	11.69 ± 3.25	8.33 ± 1.89	15.01 ± 3.02
100	22.71 ± 2.54	14.14 ± 1.39	7.99 ± 1.78	17.78 ± 1.89

^1^ The inhibition (%) of biochanin A against TACE, trypsin, chymotrypsin, and elastase is expressed as mean ± SE based on three independent experiments; ^2^ Comparison of concentration level in biochanin A is not significantly different.

## References

[1] Jakob-Roetne R., Jacobsen H. (2009). Alzheimer’s Disease: From pathology to therapeutic approaches. Angew. Chem. Int. Ed. Engl..

[2] Hardy J.A., Higgins G.A. (1992). Alzheimer’s disease: The amyloid cascade hypothesis. Science.

[3] Citron M. (2004). β-Secretase Inhibition for the Treatment of Alzheimer’s Disease Promise and challenge. Trends Pharmacol. Sci..

[4] Shoji M., Golde T.E., Ghiso J., Cheung T.T., Estus S., Shaffer L.M., Cai X.D., Mckay D.M., Tintner R., Frangione B. (1992). Production of the Alzheimer amyloid beta protein by normal proteolytic processing. Science.

[5] Urbanc B., Cruz L., Buldyrev S.V., Havlin S., Irizarry M.C., Stanley H.E., Hyman H.T. (1999). Dynamics of plaque formation in Alzheimer’s disease. Biophys. J..

[6] Holloway M.K., Hunt P., McGaughey G.B. (2009). Structure and modeling in the design of β- and γ-secretase inhibitors. Drug Dev. Res..

[7] Cai M., Wang Y., McCarthy D., Wen H., Borchelt D.R., Price D.L., Wong P.C. (2001). BACE1 is the major beta-secretase for generation of abeta peptides by neurons. Nat. Neurosci..

[8] Luo Y. (2001). Ginkgo Biloba Neuroprotection: Therapeutic implications in Alzheimer’s disease. J. Alzheimer’s Dis..

[9] McConlogue L., Buttini M., Anderson J.P., Brigham E.F., Chen K.S., Freedman S.B., Games D., Johnson-Wood K., Lee M., Zeller M. (2007). Partial reduction of BACE has dramatic effects on Alzheimer plaque and synaptic pathology in APP transgenic mice. J. Biol. Chem..

[10] Rabe S., Reichwald J., Ammaturo D., de Strooper B., Saftig P., Neumann U., Staufenbiel M. (2011). The Swedish APP mutation alters the effect of genetically reduced BACE1 expression on the APP processing. J. Neurochem..

[11] Tian X.Y., Zhao Y., Yu S.S., Fang W.S. (2010). BACE1 (beta-secretase) inhibitory phenolic acids and a novel sesquiterpenoid from *Homalomena occulta*. Chem. Biodivers..

[12] Saviranta N.M.M., Anttonen M.J., von Wright A., Karjalainen R.O. (2008). Red clover (*Trifoliumpratense* L.) isoflavones: Determination of concentrations by plant stage, flower colour, plant part and cultivar. J. Sci. Food Agric..

[13] Schrepfer S., Deuse T., Münzel T., Schäfer H., Braendle W., Reichenspurner H. (2006). The selective estrogen receptor-beta agonist biochanin A shows vasculoprotective effects without uterotrophic activity. Menopause.

[14] Tan J.W., Kim M.K. (2016). Neuroprotective effects of Biochanin A against β-amyloid-induced neurotoxicity in PC12 cells via a mitochondrial-dependent apoptosis pathway. Molecules.

[15] Chen H.Q., Jin Z.Y., Li G.H. (2007). Biochanin A protects dopaminergic neurons against lipopolysaccharide-induced damage through inhibition of microglia activation and proinflammatory factors generation. Neurosci. Lett..

[16] Occhiuto F., Palumbo D.R., Samperi S., Zangla G., Pino A., De Pasquale R., Circosta C. (2009). The isoflavones mixture from *Trifoliumpratense* L. Protects HCN 1-A neurons from oxidative stress. Phytother. Res..

[17] Biradar S.M., Joshi H., Chheda T.K. (2014). Biochanin-A ameliorates behavioural and neurochemical derangements in cognitive-deficit mice for the betterment of Alzheimer’s Disease. Hum. Exp. Toxicol..

[18] Youn K., Lee J., Yun E.Y., Ho C.T., Karwe M.V., Jeong W.S., Jun M. (2014). Biological evaluation and in silico docking study of γ-linolenic acid as a potential BACE1 inhibitor. J. Funct. Foods.

[19] Marvin 5.11.4, 2012, ChemAxon. http://www.chemaxon.com.

[20] Trott O., Olson A.J. (2010). AutoDock Vina: Improving the speed and accuracy of docking with a new scoring function, efficient optimization, and multithreading. J. Comput. Chem..

[21] Cornish-Bowden A. (1974). A simple graphical method for determining the inhibition constants of mixed, uncompetitive and non-competitive inhibitors. Biochem. J..

[22] Mahringer A., Karamustafa S., Klotz D., Kahl S., Konkimalla V.B., Wang Y., Wang J., Liu H.Y., Boechzelt H., Hao X. (2010). Inhibition of P-glycoprotein at the blood-brain barrier by phytochemicals derived from traditional Chinese medicine. Cancer Genom. Proteom..

[23] Ghosh A.K., Brindisi M., Tang J. (2012). Developing β-secretase inhibitors for treatment of Alzheimer’s disease. J. Neurochem..

[24] Zhang S., Morris M.E. (2003). Effect of the flavonoids biochanin A and silymarin on the P-glycoprotein-mediated transport of digoxin and vinblastine in human intestinal Caco-2 cells. Pharm. Res..

[25] Zhang S., Morris M.E. (2003). Effects of the flavonoids biochanin A, morin, phloretin and silymarin on P-glycoprotein-mediated transport. J. Pharmacol. Exp. Ther..

[26] Sehm T., Fan Z., Weiss R., Schwarz M., Engelhorn T., Hore N., Doerfler A., Buchfelder M., Eyüpoglu I.Y., Savaskan N.E. (2014). The impact of dietary isoflavonoids on malignant brain tumors. Cancer Med..

[27] Setchell K.D., Brown N.M., Desai P., Zimmer-Nechemias L., Wolfe B.E., Brashear W.T., Kirschner A.S., Cassidy A., Heubi J.E. (2001). Bioavailablity of Pure Isoflavones in Healthy Humans and Analysis of Commercial Soy Isoflavone Supplements. J. Nutr..

[28] Moon Y.J., Sagawa K., Frederick K., Zhang S., Morris M.E. (2006). Pharmacokinetics and bioavailability of the isoflavone biochanin A in rats. AAPS J..

[29] Moon Y.J., Morris M.E. (2007). Pharmacokinetics and bioavailability of the bioflavonoid biochanin A: Effects of quercetin and EGCG on biochanin A disposition in rats. Mol. Pharm..

[30] Yanagihara K., Ito A., Toge T., Numoto M. (1993). Antiproliferative effects of isoflavones on human cancer cell lines established from the gasatrointestinal tract. Cancer Res..

